# Large-scale next generation sequencing based analysis of *SLCO1B1* pharmacogenetics variants in the Saudi population

**DOI:** 10.1186/s40246-024-00594-9

**Published:** 2024-03-25

**Authors:** Ewa Goljan, Mohammed Abouelhoda, Asma Tahir, Mohamed ElKalioby, Brian Meyer, Dorota Monies

**Affiliations:** 1 Clinical Genomics, Centre for Genomic Medicine, King Faisal Specialist Hospital and Research Centre, P.O. Box 3354, Riyadh, 11211 Saudi Arabia; 2 Computational Biosciences, Centre for Genomic Medicine, King Faisal Specialist Hospital and Research Centre, Riyadh, Saudi Arabia

**Keywords:** Pharmacogenetics, *SLCO1B1*, Diplotypes, Phenotypes, Frequencies, Novel variants

## Abstract

**Background:**

SLCO1B1 plays an important role in mediating hepatic clearance of many different drugs including statins, angiotensin-converting enzyme inhibitors, chemotherapeutic agents and antibiotics. Several variants in *SLCO1B1* have been shown to have a clinically significant impact, in relation to efficacy of these medications. This study provides a comprehensive overview of *SLCO1B1* variation in Saudi individuals, one of the largest Arab populations in the Middle East.

**Methods:**

The dataset of 11,889 (9,961 exomes and 1,928 pharmacogenetic gene panel) Saudi nationals, was used to determine the presence and frequencies of *SLCO1B1* variants, as described by the Clinical Pharmacogenetic Implementation Consortium (CPIC).

**Results:**

We identified 141 previously described SNPs, of which rs2306283 (50%) and rs4149056 (28%), were the most common. In addition, we observed six alleles [*15 (24.7%) followed by *20 (8.04%), *14 (5.86%), *5 (3.84%), *31 (0.21%) and *9 (0.03%)] predicted to be clinically actionable. Allele diplotype to phenotype conversion revealed 41 OATP1B1 diplotypes. We estimated the burden of rare, and novel predicted deleterious variants, resulting from 17 such alterations.

**Conclusions:**

The data we present, from one of the largest Arab cohorts studied to date, provides the most comprehensive overview of *SLCO1B1* variants, and the subsequent OATP1B1 activity of this ethnic group, which thus far remains relatively underrepresented in available international genomic databases. We believe that the presented data provides a basis for further clinical investigations and the application of personalized statin drug therapy guidance in Arabs.

**Supplementary Information:**

The online version contains supplementary material available at 10.1186/s40246-024-00594-9.

## Background

Pharmacogenomics (PGx) has made important advances in recent years and is now being rapidly translated to clinical care. Organic anion transporting polypeptide (OATP) transporters, encoded by the *SLCO* gene family, are involved in absorption, distribution, metabolism and excretion of many drugs. They are crucial in drug transport across the cellular intestinal membranes, renal and pulmonary epithelial cells, brain capillary endothelial cells, hepatocytes and cancer cells [1, 2]. SLCO1B1 plays an important role in mediating hepatic clearance of many different drugs including statins such as simvastatin, angiotensin-converting enzyme inhibitors such as enalapril, chemotherapeutic agents, antibiotics such as rifampicin, the endothelin receptor antagonist besentan, the prostacyclin beraprost, the anti-inflammatory drug diclofenac, and the antiviral simeprevir [3, 4].

Genetic variations can have a profound impact on how an individual responds to drugs. Knowledge of such variation in a patient may facilitate better therapeutic options leading to better outcomes, and in some instances, preventing adverse drug events (ADE’s). For instance, simvastatin which is commonly used in the treatment of hypercholesterolemia, one of the most significant risk factors in cardiovascular disease, may lead to statin-induced myopathy (SIM) [5, 6]. Clinical trials observed that some patients on statins achieved similar benefits at lower doses when compared to others [7, 8]. The differences in drug efficacy highlight the current trend towards individualized pharmacotherapy, such that the right drug is delivered at the right dose for the right patient. A standard dose of a given drug is not always safe, effective or economical in an individual patient.

The SLCO1B1 locus (GRCh37/hg19) (chr12:21,284,128 − 21,392,730) includes many single-nucleotide polymorphisms (SNPs) including a few having significant functional effects [9]. Among these, a common variant (c.521T > C:p.Val174Ala), rs4149056, is contained within the SLCO1B1*5, *15 and *17 haplotypes. The C, or minor allele of this locus has been associated with a decrease in the SLCO1B1 transporter function resulting in reduced clearance of several drugs in vivo [9–11] and clearly links the SLCO1B1*5, *15 and *17 haplotypes with simvastatin-induced myopathy [6, 10, 11]. In patients with SLCO1B1*5, *15 and *17 haplotypes, if optimal efficacy is not achieved with a lower dose of simvastatin, alternative therapies should be considered [9, 10].

Allelic frequencies of genes encoding drug-metabolizing enzymes including *SLCO1B1* may vary considerably between ethnic groups, with incidence of SLCO1B1*5, *15 and *17 haplotypes being well documented in Caucasians and in some other ethnicities [11–13], but to date, poorly in Arabs. In this regard, this study will provide new pharmacogenetic information, specific for one of the largest Arab populations in the Middle East. Identification of known and novel allele frequencies in the Saudi population, and their phenotypic designation, will provide the basis for better clinical management of hypercholesterolemia, in Saudi Arabia and beyond.

## Results

**Fig. 1:**
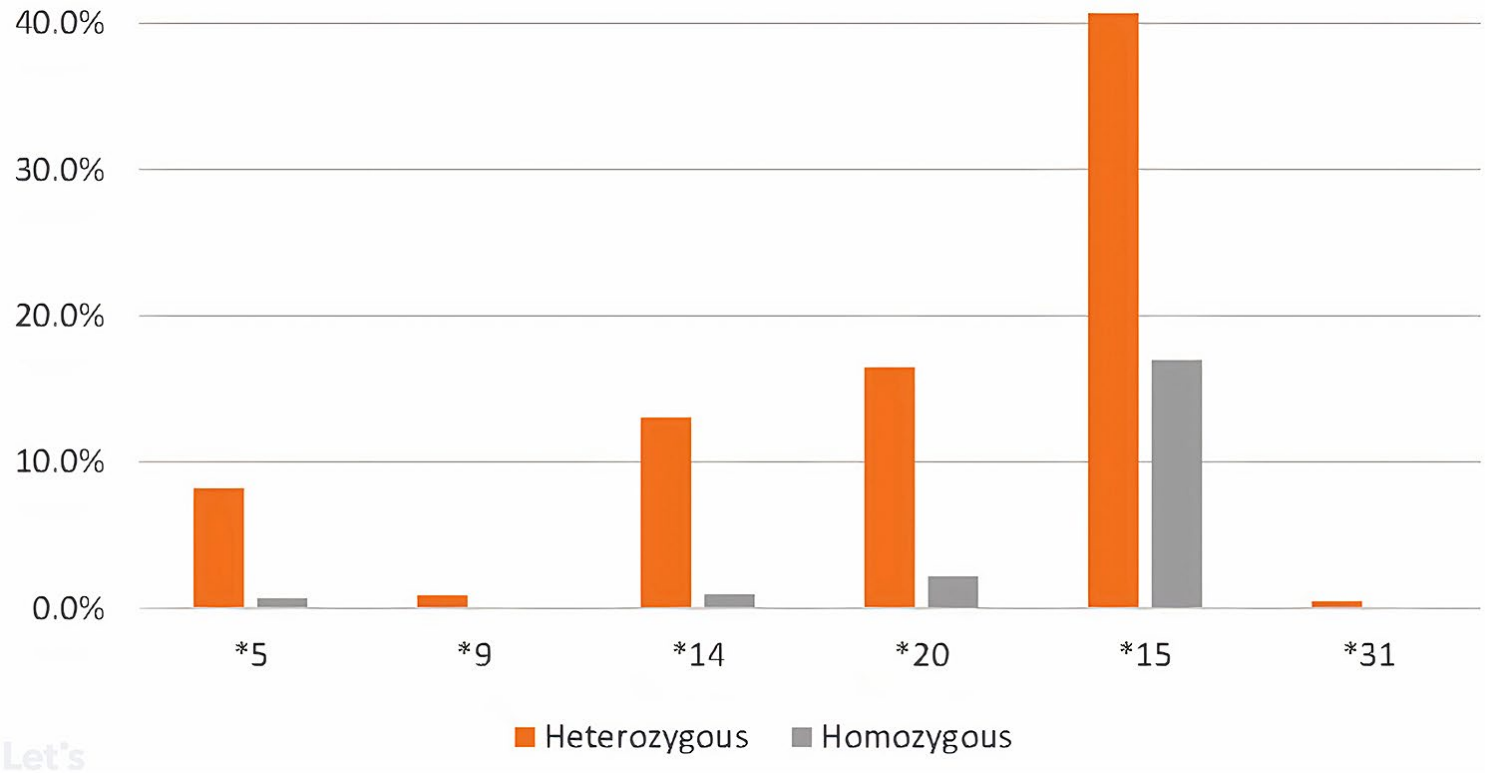
Frequency of clinically actionable *SLCO1B1* alleles in the Saudi population

We performed a comprehensive analysis of 141 *SLCO1B1* pharmacogenetic variants (Table S2) described by the Clinical Pharmacogenetic Implementation Consortium (CPIC), in 11,889 unrelated individuals from the Saudi population. This was imputed from sequence data, either a pharmacogenomic gene panel (*n* = 1,928) or whole exomes (*n* = 9,961), of individuals from this population. We identified 141 previously described SNPs of which rs2306283 (contained within SLCO1B1*15) and rs4149056 (primarily contained within the SLCO1B1*5, *15 and *17) were the most common: 50% and 28%, respectively (Table S2). Table S2 provides a comprehensive summary of functional *SLCO1B1* star allele frequencies in the Saudi population and the incidence of these in other populations, including six alleles predicted to be clinically actionable as classified by CPIC (https://cpicpgx.org/guidelines/cpic-guideline-for-statins/). Among clinically actionable alleles the most frequent were *15 (24.7%) followed by *20 (8.04%), *14 (5.86%), *5 (3.84%), *31 (0.21%) and *9 (0.03%) and in each instance represented a combination of homozygous and heterozygous individuals (Fig. 1). Allele diplotype to phenotype conversion revealed 41 diplotypes: normal function (7), decreased function (13), poor function (4), increased function (3) and indeterminate function (14) (Table 1).

**Table 1:** Frequencies of *SLCO1B1* diplotypes and their associated CPIC phenotypes in the Saudi population

SLCO1B1 Diplotype	SLCO1B1 Phenotype*	Total (*n* = 1928) Percentage of the population
*1/*1	Normal Function	18.05%
*1/*14	Normal Function	5.50%
*1/*20	Normal Function	7.26%
*1/*37	Normal Function	9.96%
*14/*37	Normal Function	1.82%
*20/*37	Normal Function	2.44%
*37/*37	Normal Function	3.16%
*1/*5	Decreased Function	3.68%
*1/*15	Decreased Function	21.32%
*1/*31	Decreased Function	0.21%
*5/*14	Decreased Function	0.36%
*5/*20	Decreased Function	0.05%
*5/*37	Decreased Function	1.04%
*9/*20	Decreased Function	0.05%
*14/*15	Decreased Function	2.65%
*14/*31	Decreased Function	0.05%
*15/*20	Decreased Function	3.48%
*15/*37	Decreased Function	5.13%
*20/*31	Decreased Function	0.05%
*31/*37	Decreased Function	0.05%
*5/*5	Poor Function	0.26%
*5/*15	Poor Function	1.87%
*5/*31	Poor Function	0.05%
*15/*15	Poor Function	7.31%
*14/*14	Increased Function	0.41%
*14/*20	Increased Function	0.83%
*20/*20	Increased Function	1.24%
*1/*19	Indeterminate Function	0.41%
*1/*28	Indeterminate Function	0.05%
*1/*30	Indeterminate Function	0.10%
*1/*41	Indeterminate Function	0.21%
*4/*37	Indeterminate Function	0.26%
*5/*19	Indeterminate Function	0.05%
*5/*41	Indeterminate Function	0.05%
*14/*19	Indeterminate Function	0.05%
*15/*19	Indeterminate Function	0.21%
*15/*41	Indeterminate Function	0.21%
*19/*19	Indeterminate Function	0.10%
*19/*37	Indeterminate Function	0.10%
*20/*41	Indeterminate Function	0.05%
*37/*41	Indeterminate Function	0.16%

The frequency of the most common diplotype *1/15 (21.32%) is similar to that in two other populations (Americans and Near Eastern). Among high frequency diplotypes, *1/37 is much less commonin Saudi individuals relative to American, European, Asians, Near Eastern and African populations (*p* < 0.05). Diplotype *1/14 (5.50%) has been previously reported in the European population and no other ethnic groups (Table S3). Predicted actionable phenotypes (poor function: 9.5% and decreased function: 38.1%) were observed in 47.6% of the Saudi population. SLCO1B1 phenotypes and their frequencies in comparison to other ethnicities are based upon CPIC data as presented in Table 2 (https://cpicpgx.org/guidelines/cpic-guideline-for-statins/).

**Table 2:** Frequencies of SLCO1B1 phenotypes in the Saudi population and other ethnicities

Phenotype	SA	AMFC	AMR	CSA	EUR	QAR
Possible Decreased Function	0	0	0	0.000042	0.000298	NA
Poor Function	0.094917	0.0001	0.0576	0.004864	0.029131	0.056392
Normal Function	0.481846	0.9801	0.5776	0.864822	0.656414	NA
Indeterminate	0.020228	0	0	0.000558	0.001445	NA
Increased Function	0.024896	0	0	0	0.029916	NA
Decreased Function	0.381224	0.0198	0.3648	0.129714	0.282796	0.26724

Saudi Arabian (SA) African American/Afro-Carribean (AMFC) American (AMR) Central/South Asian (CSA) European (EUR) Qatari (QAR)

**Fig. 2:**
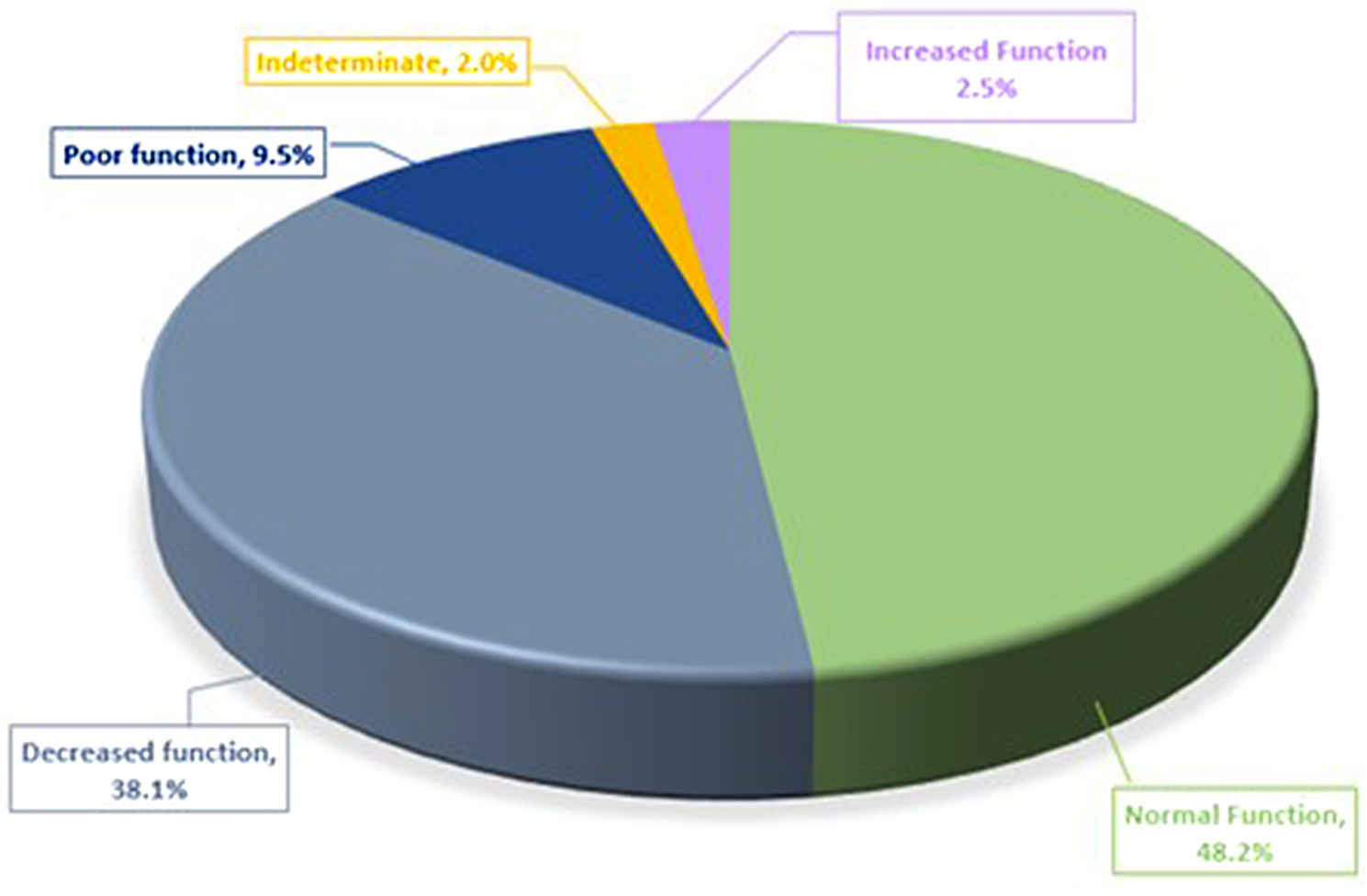
Pie chart presenting the percentage of *SLCO1B1* phenotypes found in the Saudi population

Diplotypes associated with an increased function phenotype were identified in 2.5% of our study population (Fig. 2, Table S4). We estimated the burden of rare and novel predicted deleterious variants, to have an aggregated frequency of 2.3% resulting from 17 alterations. They included 1 stop-gain, 4 splice donor, and 12 missense variants, with an ADME score calculated as described in the methods, of ≥ 80% (Table 3).

**Table 3:** Novel and rare *SLCO1B1* variants in Saudi population

Variant		Variant type	Minor allele frequency, SA (%)	
NM_006446.5:exon13:c.1706 C > A:p.Ser569Ter (novel)		stop gain	0.004	
NM_006446.5:exon11:c.1423G > C:p.Gly475Arg (novel)		missense	0.025	
NM_006446.5:exon11:c.1463G > T:p.Gly488Val (novel)		missense	0.130	
NM_006446.5:c.116T > A:p.Ile39Asn (novel)		missense	0.008	
NM_006446.5:c.84 + 1G > A		splice donor	0.004	
NM_006446.5:c.226 + 1G > A		splice donor	0.004	
NM_006446.5:c.1135 + 1G > A		splice donor	0.021	
NM_006446.5:c.1865 + 1G > A		splice donor	0.172	
NM_006446.5:c.152 C > T:p.Ser51Phe		missense	1.825	
NM_006446.5:c.170G > A:p.Arg57Gln		missense	0.008	
NM_006446.5:c.703G > A:p.Val235Met		missense	0.013	
NM_006446.5:c.1457T > C:p.Leu486Pro		missense	0.004	
NM_006446.5:c.1508 A > G:p.Asn503Ser		missense	0.004	
NM_006446.5:c.1651G > A:p.Gly551Arg		missense	0.004	
NM_006446.5:c.1784T > C:p.Ile595Thr		missense	0.004	
NM_006446.5:c.1837T > C:p.Cys613Arg		missense	0.008	
NM_006446.5:c.1841G > T:p.Arg614Met		missense	0.017	

**Fig. 3:**
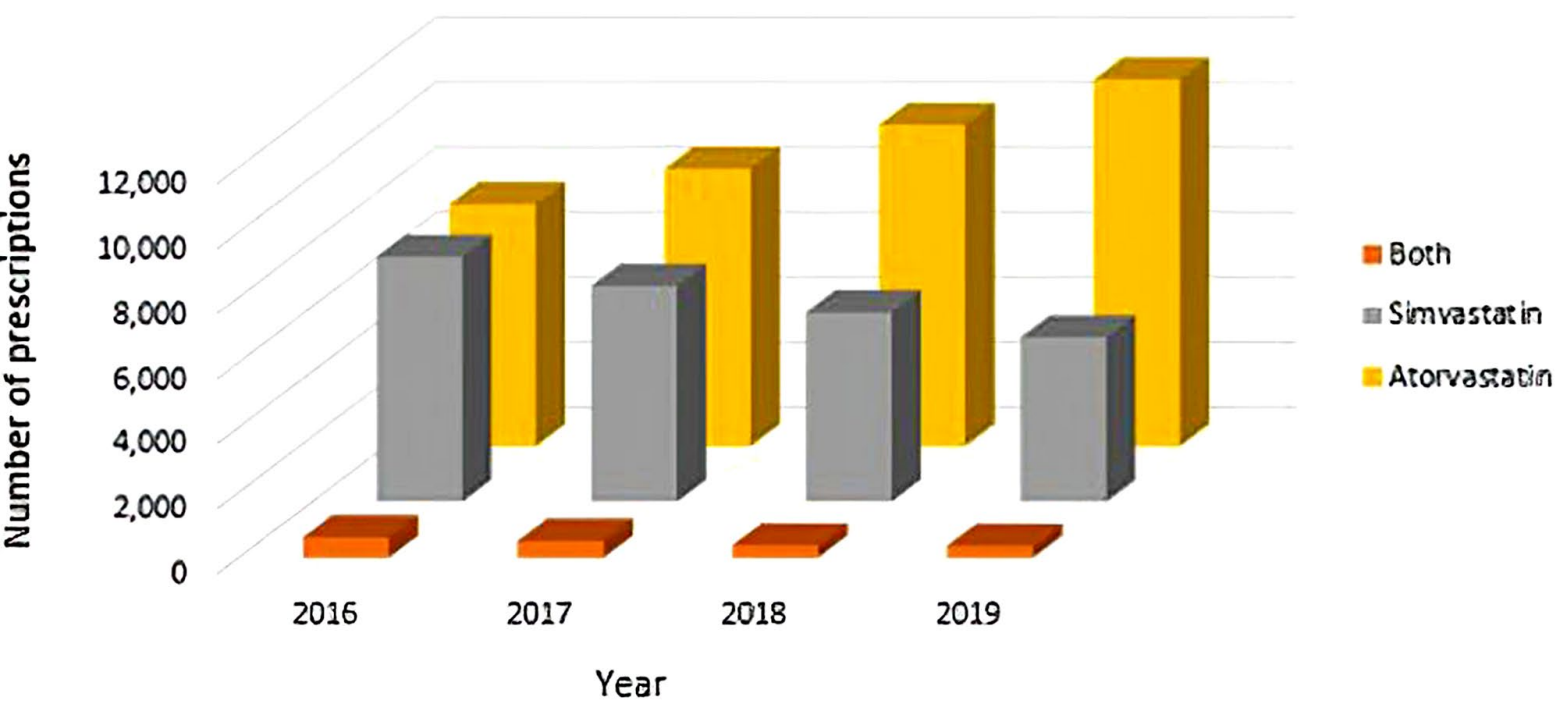
Number of simvastatin and atorvastatin prescriptions in 2016–2019 at KFSHRC

The potential for genotype-based prescription of statins is indicated by historical data from King Faisal Specialist Hospital and Research Centre (KFSHRC). At KFSHRC,from 2016 to 2019, a total of 273,167 statin drugs (simvastatin and atorvastatin) had been received by 64,034 patients. We identified three groups of patients: those who had received only simvastatin, only atorvastatin and both statins (Table 4). Proportions of simvastatin and atorvastatin prescribed for patients at KFSHRC changed between 2016 and 2019. In 2016 simvastatin was more frequently prescribed when compared to atorvastatin (7534 vs.7436) whereas from 2017 this trend had changed and atorvastatin became the more frequent medication than simvastatin with a ratio of ~ 2:1 in 2019 (Fig. 3). While statin induced toxicity may largely be resolved at KFSHRC, genotype-based statin prescription may be beneficial in the larger Saudi population.

**Table 4:** Number of patients and prescriptions for simvastatin and atorvastatin at KFSHRC in 2016–2019

Year	Number of patients per drug			Total no of patients (Simvastatin + Atorvastatin + Both)	Total no of prescriptions (Simvastatin + Atorvastatin + Both)
	Simvastatin	Atorvastatin	Both		
2016	7534	7436	619	15,589	72,152
2017	6638	8523	515	15,676	66,520
2018	5830	9876	375	16,081	64,973
2019	5060	11,270	358	16,688	69,522

## Discussion

Variants of the *SLCO1B1* transporter impact the hepatic uptake of statins and a large number of endogenous substrates. The most common statin-related adverse drug reaction (ADR) is skeletal muscle toxicity that results in statin-associated musculoskeletal symptoms (SAMS) [14, 15]. In 2008, data from a big genome-wide study showed a strong association between a *SLCO1B1* variant (rs4149056) and an increased risk of statin-induced myopathy. This finding was further confirmed by additional studies that revealed an odds ratio (OR) of 2.3 and 3.2, for myopathy in subjects exposed to simvastatin, with the CT and CC genotypes of rs4149056, respectively [16]. This finding has since been replicated in an independent trial and practice-based longitudinal cohort of 20,000 subjects [17]. In 2012, the Clinical Pharmacogenetics Implementation Consortium (CPIC) released gene-based prescribing guidelines for simvastatin based on *SLCO1B1* genotypes [18], updated further in 2014 [10] and again in 2022 [19]. They provided therapeutic recommendations for statins with the goal of improving the overall safety, adherence and effectiveness of statin therapy. Individualized pharmacotherapy using the right drug and at the right dose is very critical to optimal outcomes in patients on statins. Allele frequencies of *SLCO1B1* vary across multiple ancestries and geographically diverse groups (https://cpicpgx.org/guidelines/cpic-guideline-for-statins/). To the best of our knowledge, this is the first study of *SLCO1B1* polymorphism in the population of Saudi Arabia, a population not well represented in current databases. Mining of large-scale NGS data is a very powerful tool for cataloguing the range and frequency of genetic variation in populations [20]. Many SNPs have been identified in *SLCO1B1* but thus far, only a few are known to have functional effects [14, 21]. We found that the common C allele at (c.521T > C) of rs4149056 (*SLCO1B1* *5) and the *SLCO1B1* *15 alleles associated with lower plasma clearance of simvastatin was present with higher frequency in the Saudi population when compared to other ethnic groups (Table S2). The high frequency of the c.521T > C allele (28%) results in the higher frequency of *allele diplotypes based on which SLCO1B1 phenotypes are assigned.

Predicted actionable diplotypes (decreased and poor function), that are associated with an increased or high risk of myopathy were strongly represented in our study population. Their frequency was higher than seen in another Middle East population from Qatar (47.61% versus 32.37% respectively). It was also much higher compared to European, East Asian, Near Eastern and American populations (~ 29%), and thus far, the highest observed in the CPIC database (Table S4). We can estimate that ~ 12,000 (47.61%) patients who received simvastatin at KFSHRC from 2016 to 2019 are phenotypically either decreased or poor function, with respect to SLCO1B1 activity, and would potentially benefit from personalized simvastatin dosing or the use of alternate therapies. At KFSHRC, we observed a trend between 2016 and 2019 of simvastatin being replaced by atorvastatin. It may reflect toxicity or poor tolerance of simvastatin. Our study, as well as data from other Middle East populations [22], suggest that Arabs may be at greater risk of simvastatin induced myopathy. Since there are alternate hypolipidemic drugs, that have a lower substrate affinity for OATP1B, they may be alternatives for patients intolerant of statins. Implementation of *SLCO1B1*genotyping before statins are prescribed [19], could be very helpful in Arabs. The current clinical prediction of SLCO1B1 phenotypes is developed based on studies conducted mainly in Europeans and Americans. To the best of our knowledge, such studies have never been performed in Arabs. It is well known that simvastatin-related muscle toxicity still occurs in patients with WT alleles of rs4149056 and we cannot thus exclude the possibility of other potentially deleterious variants in *SLCO1B1* closely linked to rs4149056 [18]. The high number of individuals who carry actionable *SLCO1B1* dilpotypes, strongly support further studies in Middle Eastern populations.Individuals who have developed clinically and laboratory confirmed SAMS, should be further investigated to identify novel *SLCO1B1* variants, that have clinical significance. Such studies in the Arab population have substantial potential to discover new variants that underlie statin-related myopathy.

By leveraging large-scale whole-exome sequencing data from Saudi individuals, we for the first time, present data on novel, and predicted deleterious *SLCO1B1* variants (Table S1 and Table S5) in Arabs. At present, pharmacogenetic testing is performed mainly by SNP arrays targeting specific alleles, and hence, the detection of rare and novel variants is not possible. Unexplained statin toxicity should not be neglected and should be studied further, as rare or novel variants may account for nearly all inter-individual variabilities in pharmacogenes [23]. Rare deleterious variants with an aggregated frequency of 1.2%, jointly explained 8.7% of the genetic basis of *SLCO1B1* variability in other studies [24]. Considering the high frequency (2.3%), of rare and novel predicted pathogenic alterations, detected by this study in the Saudi population, it is important to determine their clinical impact. Notably, the nonsense c.1706 C > A:p.S569X with a homozygous frequency of 0.03% should be further investigated.

## Conclusions

Our study highlights the value of mining large NGS databases, as being a powerful tool, to improve knowledge of genomic variation in clinically relevant genes. The data we present from one of the largest Middle Eastern countries, provides the most comprehensive overview of *SLCO1B1* variants, and the subsequent OATP1B1 activity in a large cohort of Arabs, a population still underrepresented in available international genomic databases. We believe studies such as this, provide important information for the guidance of personalized drug therapies, in Arab patients.

### Methods

The dataset used for mining of *SLCO1B1* variants comprised 9,961 exomes and 1,928 PGx custom gene panels (genes are listed in Table S1), from unrelated Arab individuals, sequenced by the Saudi Human Genome Program (SHGP) between 2015 and 2019, as part of a comprehensive investigation of rare diseases in the Saudi population [25, 26]. The Clinical Pharmacogenetics Implementation Consortium (CPIC) guidelines (https://cpicpgx.org/guidelines/) for *SLCO1B1* and simvastatin-induced myopathy were used to assign the likely OATP1B1 phenotypes: normal function, decreased function, poor function, increased function, indeterminate function and *allele nomenclature [10]. We used Stargazer software (v.1.0.8.5) to compute star alleles. This algorithm performs statistical haplotype phasing using Beagle [27] with reference samples from the 1000 Genomes Project [28]. The Beagle method is based on a localized haplotype-cluster model, which is an empirical linkage disequilibrium model, that can take the local structure of data into consideration. The Beagle algorithm is accurate and fast due to the use of an EM-based algorithm that literately fits the best model to the data [27]. Afterwards, the phased haplotypes computed by Beagle are matched to publicly available star allele information, mostly in the PharmVar database (https://www.pharmvar.org) and PharmGKB (https://www.pharmgkb.org/). Finally, Stargazer reports the star allele findings in a tabular format, along with prediction of the related metabolizer information.

Variants with MAF < 1% were defined as rare and genetic alterations with frequencies that exceeded the observed frequencies in other populations (European, Finish, Hispanic, African, South Asian, East Asian and Ashkenazi Jews) by > 20-fold were considered as being unique to the Saudi population. Next, we classified alleles as novel if they were not observed in: 1000 Genomes (phase3), gnomAD (v.3.1.1), Exac (v.0.3) and Kaviar (v.160,204). Functional consequence of PGx, rare Saudi-specific, and novel variants were predicted using a two-fold approach. Any variants with a high IMPACT rating, such as frameshift indels or stop loss variants, were considered to be deleterious [29]. We then applied the ADME (chemical absorption, distribution, metabolism, excretion, and toxicity) optimized framework, that is an ensemble of deleteriousness prediction methods, for predicting deleteriousness in pharmacogenes. We used 18 prediction algorithms including CADD, SIFT, PolyPhen, LRT (likelihood ratio test), MutationAssessor, FATHMM, FATHMM-MKL, PROVEAN, VEST3, DANN, MetSVM, MetaLR, GERP++, SiPhy, PhyloP-vertebrate, PhyloP-mammalian, PhastCons-vertebrate, and PhastCons-mammalian to compute the ADME scores. ADME scores larger than 80% were considered to affect pharmacogene functionality [30].

### Electronic supplementary material

Below is the link to the electronic supplementary material.

Additional file 1: Table S1 List of PGx gene panel.

Additional file 2: Table S2 List of previously described PGx SNPs.

Supplementary Material 3

Additional file 4: Table S4 Frequency of SLCO1B phenotypes.

Additional file 5: Table S5 List of predicted deleterious SLCO1B1 variants.

## Data Availability

National regulations prevent uploading of genomic data to sites outside Saudi Arabia. Requests to access these datasets should be directed to mabouelhoda@kfshrc.edu.sa.

## Abbreviations

PGx: Pharmacogenomics
SAMS: Statin-associated musculoskeletal symptoms
ADR: Adverse drug reaction
CPIC: Clinical Pharmacogenetic Implementation Consortium
OATP: Organic anion transporting polypeptide
SHGP: Saudi Human Genome Program
MAF: Minor Allele Frequency
ADME: Chemical absorption, distribution, metabolism, excretion, and toxicity
KFSHRC: King Faisal Specialist Hospital and Research Centre
WT: Wild Type
NGS: Next Generation Sequencing
